# A pilot cluster randomised trial to assess the effect of a structured communication approach on quality of life in secure mental health settings: The Comquol Study

**DOI:** 10.1186/s12888-016-1046-8

**Published:** 2016-09-29

**Authors:** Douglas MacInnes, Catherine Kinane, Janet Parrott, Jacqueline Mansfield, Tom Craig, Sandra Eldridge, Ian Marsh, Claire Chan, Natalia Hounsome, George Harrison, Stefan Priebe

**Affiliations:** 1 Canterbury Christ Church University, Canterbury, UK; 2 Kent and Medway NHS and Social Care Partnership Trust, Maidstone, UK; 3 Oxleas NHS Foundation Trust, Dartford, UK; 4 Institute of Psychiatry, Kings College London, London, UK; 5 Queen Mary University of London, London, UK

**Keywords:** Comquol, DIALOG, Forensic, Mental health, Quality of life, Solution focused brief therapy, Service user collaboration

## Abstract

**Background:**

There is a lack of research in forensic settings examining therapeutic relationships. A structured communication approach, placing patients’ perspectives at the heart of discussions about their care, was used to improve patients’ quality of life in secure settings.

The objectives were to:

• Establish the feasibility of the trial design

• Determine the variability of the outcomes of interest

• Estimate the costs of the intervention

• If necessary, refine the intervention

**Methods:**

A pilot cluster randomised controlled trial was conducted. Data was collected from July 2012 to January 2015 from participants in 6 medium secure in–patient services in London and Southern England. 55 patients and 47 nurses were in the intervention group with 57 patients and 45 nurses in the control group. The intervention comprised 6 nurse-patient meetings over a 6 month period. Patients rated their satisfaction with a range of domains followed by discussions on improving patient identified problems. Assessments took place at baseline, 6 months, and 12 months. Participants were not blind to their allocated group. The primary outcome was self-reported quality of life collected by a researcher blind to participants’ allocation status.

**Results:**

The randomisation procedures and intervention approach functioned well. The measures used were understood by the participants and gave relevant outcome information. The response rates were good with low patient withdrawal rates. The quality of life estimated treatment effect was 0.2 (95 % CI: −0.4 to 0.8) at 6 months and 0.4 (95 % CI: −0.3 to 1.1) indicating the likely extreme boundaries of effect in the main trial. The estimated treatment effect of the primary outcome is clinically important, and a positive effect of the intervention is not ruled out. The estimate of the ICC for the primary outcome at 6 and 12 months was 0.04 (0.00 to 0.17) and 0.05 (0.00 to 0.18). The cost of the intervention was £529 per patient.

**Conclusions:**

The trial design was viable as the basis for a full-scale trial. A full trial is justified to estimate the effect of the intervention with greater certainty. The variability of the outcomes could be used to calculate numbers needed for a full-scale trial. Ratings of need for therapeutic security may be useful in any future study.

**Trial registration:**

Current Controlled Trials ISRCTN34145189. Retrospectively registered 22 June 2012.

## Background

Forensic mental health care is the provision of mental health services for people with mental disorders who are offenders or at risk of offending. Services are provided in secure, community, NHS and criminal justice settings [1]. Many of these patients are managed in medium secure inpatient facilities. Medium secure facilities are designed to provide higher physical, procedural and relational security measures than an ordinary hospital ward or a low secure ward but less so than in the handful of high security hospitals in the UK. Patients in medium secure services move from admission, through rehabilitation, and towards leave and moving on. The number of beds in medium secure units has increased significantly over the last 20 years. This has been partly due to rising demand, increased length of say, and the drive to reduce high secure hospital places. It was estimated that in 2012, there were approximately 70 units in the UK with about 5,000 patient beds, and an annual national spend of £1.2bn [2]. The patients include difficult, dangerous and/or extremely vulnerable people whose behaviours present a risk to themselves as well as others. They can be difficult to engage in assessment, treatment and research and staff must meet the therapeutic needs of patients whilst addressing legal, security and public safety issues.

The Best Practice Guidelines in Medium Secure Units state the therapeutic alliance between staff and patients is at the centre of high-quality care and treatment in secure settings. This has been most often noted when discussing the importance of the term relational security which has been described as the therapeutic alliance between staff and patients in continuing risk assessment and detailed knowledge of the patient [3]. The Royal College of Psychiatrists [4] have suggested it is the most important type of security in mental health work as it achieves safety through establishing good rapport and an effective therapeutic alliance between patients and staff. Developing good therapeutic relationships also has the potential for producing clinical and social benefits so it is important to be able to ascertain the ways in which it influences service users’ perceptions of their care and treatment.

However, a review of forensic mental health services noted a lack of a patient perspective and involvement in the service [5]. The report recommended future work should seek to build mechanisms and services that involve patients and respond to their views. Research findings from non-forensic settings have also reported significantly better clinical outcomes, with reductions in unmet need, lower levels of psychopathology, higher global functioning, lower social disability, higher quality of life, and better satisfaction with services, when an agreed clinician-patient intervention strategy was in place [6, 7]. Nevertheless, there is a lack of research in forensic hospital settings concerning therapeutic relationships and no published research which examines relational security in secure settings. It has also been proposed that quality of life assessments may represent the best way of measuring the totality of detained forensic patients' experience in secure environments to guide the development and improvement of patient care [8].

Research undertaken in primary mental health care settings has indicated a patient-centred approach, including active participation of patients in the treatment process, is associated with better quality of life, increased adherence to treatment regimens and reduced misunderstanding between clinicians and patients [9, 10]. A positive relationship with the primary worker has also been consistently found to predict a better outcome in relation to symptomatology, time in hospital, and quality of life [11]. Priebe and colleagues [12] have developed an intervention using a structured communication approach called DIALOG which uses a computer-mediated approach to structure and guide the focus of the discussion between clinician and patient and places the patients’ perspective of their care at the heart of these discussions. This has been found to be an effective practical method of improving patients’ involvement in their treatment. In a trial with community patients with psychosis in six European countries, the intervention group had significantly higher quality of life scores, satisfaction with treatment, and less unmet needs, compared to the control group.

The underlying rationale of this structured communication approach is that it facilitates explicit negotiations about what each individual patient wants and what the clinician can do about it. The hypothesis presented is that this focus on the individual concerns of the patient will, in turn, lead to an improvement in subsequent care and the patient’s quality of life. It was proposed that using a structured patient-clinician communication approach using a computer-mediated approach (DIALOG) in conjunction with non-directive counselling based on the principles of Solution Focused Brief Therapy (SFBT) within a forensic mental health setting would improve patients’ quality of life, levels of satisfaction, engagement with services and reduce disturbance. There was, however, a need to pilot the intervention in this setting since DIALOG had not been tested in a forensic environment before and there was some uncertainty whether the main trial would be feasible. The specific objectives of this pilot study were to: establish the feasibility of the trial design as the basis for determining the viability of a large full-scale trial; determine the variability of the outcomes of interest; estimate the costs of the intervention; and refine the intervention following the outcome of the study based upon the experiences of the clinicians and patients.

## Methods

### Design

**Fig. 1:**
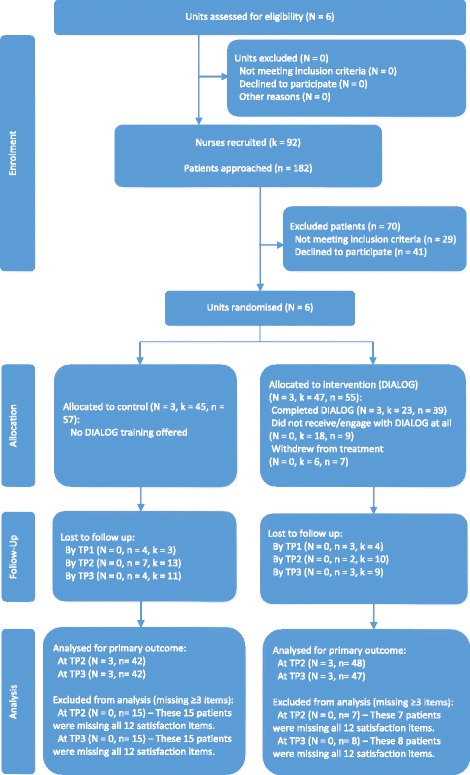
CONSORT Flow Diagram

A pragmatic cluster randomised trial was designed avoiding any potential contamination between the intervention and control groups in clinical practice. The eligibility criteria for a cluster were that they were secure mental health units with at least two medium secure wards as part of the unit. All NHS units meeting this criteria within a 50 mile radius of London were informed of the trial via email by members of the research team (DM, JP or CK) and invited to participate. The first six secure units who responded were randomised. Far fewer women than men are resident in secure units. To enable the study to examine the intervention with both men and women in the forensic mental health service, the units were stratified. The first stratum included four medium secure units with two male wards in each unit participating in the study. The second stratum consisted of two medium secure units with one male ward and one female ward in each unit participating in the study. Within both groups there was a balanced design resulting in the same number of units in each of the intervention and control groups (Fig. 1). A six-month intervention approach was developed based on the work of Priebe et al. [11, 12]. The protocol is described in fuller detail [13].

### Ethical approval

Ethical Approval was obtained from the London Surrey Borders Research Ethics Committee (reference number 11/LO/0104).

### Participants

The participants were mental health nurses and in-patients at six medium secure units in Southern England and London. The first six units that expressed an interest in being included in the study were then visited by members of the research team to discuss potential involvement in the study. This involved presentations to clinical staff detailing the aims and objectives of the study and clarifying what inclusion the study would entail for the unit team. All six units agreed to participate in the study. Agreement was undertaken through email correspondence between the senior member of the unit’s management team and the Chief Investigator. This was then followed up by a formal agreement with the relevant Research and Development manager.

The allocation was performed by the randomisation service of the registered Pragmatic Clinical Trials Unit (PCTU) at Bart’s and the London School of Medicine and Dentistry. Nurses were initially approached in two wards in each of the participating units (twelve wards overall). The inclusion criterion for the clinicians was they were registered mental health nurses working with in-patients within those wards. After nurses had been recruited to provide the intervention to a sufficient number of patients in the unit, the patients were approached. Each patient residing in participating wards was eligible to participate as long as the following inclusion criteria were met; they had a history of least 3 months of current in-patient treatment in the service and were capable of giving informed consent. Informed consent from both nurses and patients was obtained before inclusion into the study by the Research Assistant. Once recruitment in a unit had been completed, the Research Assistant emailed the PCTU with the site ID number (known only to the Research Assistant) and requested an allocation for the unit. The allocation was generated by a statistician independent of the study using the statistical software randomisation.com. An email was then sent back to the Research Assistant indicating whether the unit was allocated to the intervention or control group. To avoid bias, the random allocation of a unit into either the intervention or control arm of the study was only undertaken following the identification and recruitment of a sufficient number of nurses and patients from each unit [14] meaning the researchers were blind to allocation status at the point of nurse and patient entry into the study. All of the research team apart from the Research Assistant remained blind to the allocation status of each unit until the end of the trial. The study aim was that ten nurses, from each ward in the intervention units, would be trained in the structured communication approach, and a similar number recruited for the control group. This would allow for some drop-outs. The sample size was chosen to provide sufficient clusters in the intervention arm to provide evidence about feasibility, and sufficient individual participants and nurses to be able to judge retention rates and acceptability of the intervention. It was assumed that each ward would have approximately 16 patients, that 50 % would agree to participate and there would be a 25 % drop out. We aimed to recruit 96 patients in 12 wards in 6 clusters. A sum of £35 was given to each patient participant from both the intervention and control groups on completion of each set of assessments.

### Interventions

Participants allocated to the intervention group received the structured communication approach; the DIALOG approach combined with counselling guided by SFBT. This involved monthly meetings between the patient and nurse for a period of six months and arranged as part of routine care. The intervention consisted of two elements: a computer-mediated approach in conjunction with non-directive counselling which has been found to be an effective practical method of developing patients’ involvement in their treatment. DIALOG was used by nurses to facilitate structured communication sessions, in addition to continuing with standard treatment with their participating patients, to enable individualised therapeutic discussions. During the meeting the patients completed a simple rating checklist, recording the degree of satisfaction with eleven life and treatment domains. The domains were; mental health, physical health, accommodation, job situation, leisure activities, friendships, relationship with family/partner, personal safety, practical help, meetings and medication. Each domain was rated on a scale of 1–7 (from ‘couldn’t be worse’ to ‘couldn’t be better’), and followed by a question on whether the patient wanted any additional or different help in the given domain. If the patient answered yes, the type of the requested additional or different support was discussed and recorded. The eleven domains were presented in a fixed order and an explicit response was required for each item before proceeding to the next item. Participants’ answers to all questions were entered directly onto the iPad tablet using specifically developed software. The tablet allowed patients and nurses to view screen displays detailing the current rating of a domain as well as the rating from any previous month. The procedure was designed to ensure the patient’s views on their situation and needs for care were the central point of treatment discussions and the patient’s view on what kind of help would improve their current situation was explicit.

The counselling approach offered was Solution Focused Brief Therapy. It is a structured conversational approach that promotes movement towards positive change in individuals, families, and other systems. The approach is characterised by a focus on the future, more specifically, exploring what will be different when things are better.

A three day training programme was offered to all nurses in the intervention group to help ensure the DIALOG approach was consistently administered.

A. Each nurse in the intervention group was individually trained to use the software and provided with written instructions on how the ratings should be used to facilitate a dialogue.
B. Solution Focused Brief Therapy training was delivered by an experienced solution-focused therapist who runs a Masters course in solution focused therapy and a founder member of the UK Association of Solution Focused Practice.

Each nurse also received a practical handbook explaining how to conduct the solution focused approach to help ensure a similar approach was used in all sessions. The fidelity of the intervention was assessed and ensured through a number of procedures. The nurses brought their notes and thoughts from the initial sessions to the 2^nd^ and 3^rd^ training sessions for review by and the SFBT trainer. The nurse facilitating the sessions recorded the main topics of each session on a record sheet at the end of each session. Additionally, up to two sessions, out of the six session intervention, were audio recorded. The record sheets and recordings were reviewed by the Research Assistant (JM) who had attended the training sessions and was aware of the main principles of the SFBT approach. Monthly meetings were also held between the Research Assistant and each nurse to examine the intervention.

### Usual care

Nurses in the control arm were encouraged to meet patients with the same frequency as in the intervention group. These meetings were used to plan and evaluate care as well as to discuss any specific difficulties but without using the formalised structured communication approach.

## Assessment

### Feasibility

To assess the feasibility the following areas were considered; the recruitment process for clusters, nurses and patients, rates of completion of training, completion of outcomes, loss to follow-up, withdrawal and the number and timing of monitored sessions in each arm, and patterns of missing information.

### Outcomes

Outcome data was used to assess the potential for effectiveness and the variability in outcome measures in readiness for a sample size calculation for the main trial. The primary outcome was Quality of Life and assessed by the Manchester Short Assessment of Quality of Life scale (MANSA) [15]. It has sixteen questions with responses recorded on a seven point Likert scale. The first twelve questions are satisfaction ratings and these form the overall subjective quality of life mean scores reported in this trial. The questions were given to the participants prior to them being contacted by a researcher blind to the allocation status of the participants. The researcher interviewed the participants by phone, asking the MANSA questions, and noting down their responses.

The primary endpoint was measured at three time points. To ensure results were not affected by a secular trend, each intervention group unit was paired with a control unit group and the assessments for each paired intervention-control group unit carried out within a month of each other at each time point.

Time Point 1 - baseline assessment of patients; for the intervention group this was prior to their first structured communication session while for the control group this was at the same time as noted above;

Time Point 2 – within the two weeks following the intervention; the last structured communication approach meeting (after six months);

Time Point 3 – six months post intervention (twelve months after time point one).

The first five secondary outcomes noted below were also assessed prior to the intervention (baseline), at 6 months (post intervention) and 12 months.

### Secondary outcomes

- Engagement with Services - Helping Alliances Scale (HAS) [16]
- Ward Climate - Essen Climate Evaluation Schema (EssenCES) [17]
- Patient Satisfaction - Forensic Satisfaction Scale (FSS) [18]
- Recovery - Process of Recovery Questionnaire (QPR) [19]
- Nurse Stress - Maslach Burnout Inventory (MBI) [20]
- Disturbed behaviour was recorded from the ward untoward incident forms and patient progress notes on a monthly basis from three months prior to the baseline assessment till the six-month post intervention follow up (15 time points).

For the intervention groups only, the following outcomes were also documented:

- DIALOG Satisfaction Checklist (completed in monthly session)
- Focus groups with patients (one in each intervention unit following completion of intervention)
- Monthly interviews with nurses (completed after each session)

The patients’ demographic details, the completeness of outcomes, recruitment rates, and withdrawal rates were also recorded.

### Qualitative data

Following the final session, three focus groups were convened, one in each intervention unit, to explore service user perspectives and experiences of the study [21]. Participants were asked to give their views on the intervention and study procedures. Each group contain between four and eight patients and lasted 30–60 min. The focus groups were digitally audio-recorded and transcribed. A patient member of the research team was involved in developing the interview schedule and moderating the groups [22].

Monthly interviews were held nurses and a member of the research team (JM) from their first meeting to their final session to look at any identified concerns surrounding the intervention and to examine the acceptability of the approach from the nurse’s perspective and were also digitally audio-recorded and transcribed.

The research team coded and themed the transcriptions in line with Braun and Clarke’s thematic analysis approach [23]. Multiple coding for the focus group analysis was also employed drawing on Sweeney et al’s notion that the service user researcher unique perspective should be preserved rather than subsumed [24].

### Statistical analysis

To establish the feasibility of a full-scale cluster randomised trial the estimated treatment effect and corresponding confidence intervals for all outcomes measured at 6 and 12 months was calculated. Firstly, a mean value of the outcome for each unit was calculated, and then the treatment effects (and corresponding confidence intervals) as the mean difference of these means in the intervention and control groups. This was viewed as an acceptable method of analysing data from cluster randomised trials when the number of clusters is small. To assess the likely size required for a full scale trial some estimate of the variability of the primary outcome was needed and, in particular for a cluster randomised trial, an estimate of the intra-cluster correlation coefficient (ICC). Relying on pilot studies or even single previous trials for reliable estimates of the ICC is problematic because sampling errors are very large. Nevertheless, the randomisation units in this study are unusual and ICCs from a number of other similar studies (the safest way of calculating ICCs) were not available. Therefore, an ICC from this study was calculated, using standard analysis of variance techniques. The ICC was calculated for all primary and secondary outcomes, at 6 months and 12 months, except for disturbance monitoring and the DIALOG satisfaction checklist. Some measure of variability was also given for all primary and secondary outcomes (i.e. standard deviation, interquartile range).

In general, where there were missing data for the primary and secondary outcomes, individuals who were missing more than 20 % of the items for mean scores were excluded. For sum scores, where an individual was missing less than some pre-specified number of items, their missing item(s) were replaced with the average of the other items given, or the individual was excluded if they were missing more than the pre-specified number of items for that particular outcome.

### Economic costs

The health economics evaluation adopted an NHS/Personal Social Services and police services perspective. Economic evaluation methods followed the NICE Guide to the Methods of Technology Appraisal 2013 [25]. A micro-costing of the DIALOG approach combined with counselling guided by SFBT included a bottom-up construction of the costs associated with setting up and delivering the intervention.

The cost of training per nurse was estimated using the number of nurses enrolled on the DIALOG-SFBT course and the number of nurses who completed the course. Sensitivity analyses considered the minimum and maximum number of nurses per session as observed in the study. The cost of the intervention per patient was estimated with and without the costs of additional staff time. Sensitivity analyses were conducted using different durations of meetings between the nurse and the patient (20, 30 and 60 min).

A micro-costing of incidents was conducted based on informed clinical opinion. The data were collected using an Incidents Resource Use Questionnaire which was designed for the study. Clinicians were asked to specify resources associated with incidents and to indicate the probability of their use. These included A&E admissions, inpatient stay, outpatient appointments, investigations, medication, staff time associated with managing incidents, NHS transport and police. The cost of an incident was calculated by multiplying the probabilities of using services (taken from the Incidents Resource Use Questionnaire) by unit costs. The total cost of incidents was derived by multiplying the number of incidents extracted from medical records by incident costs.

### Cost-consequences analysis

Costs and outcomes are presented in a disaggregated form. The cost of the intervention was calculated as per trial and presented as a total cost for the intervention and as a cost per patient. Outcomes for the cost-consequences analyses are presented per group (intervention and control) for the period from baseline assessment until the six-month post-intervention follow-up (12 months). As a pilot study, the outcomes for the intervention and control groups were not compared statistically.

## Results

### Viability of full scale trial

#### Recruitment

The research team contacted nine units about the possibility of participating. The first six who responded were contacted to discuss their involvement. All six agreed to participate. 112 patients were recruited to the study out of 182 approached in the six units (62 % recruitment rate) as shown on the flow diagram (Fig. 1). These numbers were more than the initial study aim of recruiting 96 patients. Unfortunately, there was a time delay between recruitment and starting the intervention for the first stratum cohort and, as a result, some patients withdrew from the study before undertaking the baseline assessment. This resulted in 107 patients providing baseline demographic data; 54 in the intervention group and 53 in the control group. In both groups, 85 % of the participants of the 107 giving baseline demographic data were male and 15 % women. 92 nurses were recruited to the study; 47 in the intervention group and 45 in the control group. The process for recruiting nurses to the study required discussions with each unit to identify the best way of organising the delivery of the intervention in the unit. Consequently, each unit adopted an approach that best suited its working practices. This resulted in 27 nurses being recruited in unit one with all registered nurses eligible to participate while only 5 nurses were recruited in unit six with specific nurses being identified and supported to undertake the training. Due to the delay in commencing the intervention 7 nurses in the initial cohort (unit one and unit two) did not complete baseline assessments.

There were difficulties in gaining access to some units mainly due to security concerns resulting in delays in recruiting potential participants. The most serious delay followed a major untoward incident in one of the unit’s which resulted in the research team being unable to access the unit for several months.

The process of recruiting nurses was straightforward once access to the unit was established. For the nurses taking part involvement in the study was seen as a good way of developing skills and receiving training in a new type of counselling. The patients who took part in the focus groups noted the innovative way in which the intervention was to be delivered as a motivation for taking part. The money on offer for completing the assessments was also a major incentive.

### Training

35 out of the 47 nurses (74 %) randomised to the intervention group completed the training programme. The rates for the three units varied: unit one: 18 out of 27 (67 %); unit three: 13 out of 15 (87 %); unit six: 4 out of 5 (80 %). The higher dropout rates for unit one can be partly explained by the time delays between recruitment and the start of the intervention. Some nurses had moved wards so could not pair up with patients in the wards where the intervention was being carried out. Some nurses did not attend the sessions as the patients who they had been paired with for the intervention had withdrawn from the study. In addition, some nurses from all three units decided that they did not wish to undertake the training and be part of the intervention after initially agreeing to participate. Qualitative data from the monthly meetings between the nurses and the research team as well informal discussions with the SFBT trainer indicate the nurses found the training programme stimulating and enjoyable.

### Baseline characteristics

The baseline demographic characteristics of the participants are shown in Table 1 while the baseline outcome measures are shown in Table 2.

**Table 1:** Baseline characteristics

	Intervention (*N* = 3, *n* = 55)	Control (*N* = 3, *n* = 57)
Patient Characteristics		
Age (years) – mean (SD)^a^	36 (10)	34 (11)
Gender – no. (%)^a^		
Male	46 (85)	45 (85)
Female	8 (15)	8 (15)
Ethnicity – no. (%)^a^		
Asian	4 (7)	3 (6)
Black	11 (20)	20 (38)
Mixed or Other	5 (9)	10 (19)
White	34 (63)	20 (38)
Current unit residing on – no. (%)		
Unit 1	21 (38)	0 (0)
Unit 2	0 (0)	18 (32)
Unit 3	17 (31)	0 (0)
Unit 4	0 (0)	23 (40)
Unit 5	0 (0)	16 (28)
Unit 6	17 (31)	0 (0)
Clinical diagnosis – no. (%)^b^		
Schizophrenia and Schizoaffective disorders	39 (74)	41 (79)
Other	14 (26)	11 (21)
Length of current admission – median (IQR)^a^	434 (197, 869)	554 (188, 1127)
Leave status – no. (%)^c^		
Escorted grounds/community	34 (63)	26 (50)
Unescorted grounds/community	8 (15)	9 (17)
No leave/leave for medical appointment only	12 (20)	17 (33)
MHA status – no. (%)^d^		
Section 37/41	37 (69)	34 (65)
Other	17 (31)	18 (35)

^a^Data missing for 5 patients (1 in intervention arm and 4 in control arm)

^b^Data missing for 7 patients (2 in intervention arm and 5 in control arm) (where 1 patient on control and 1 patient on intervention answered as “couldn’t rate/answer”)

^c^Data missing for 6 patients (1 in intervention arm and 5 in control arm) (where 1 patient on control answered as “couldn’t rate/answer”)

^d^Data missing for 6 patients (1 in intervention arm and 5 in control arm)

**Table 2:** Outcomes at Baseline, 6 Months and 12 Months

	Intervention clusters (*n* = 55, k = 47)			Control clusters (*n* = 57, k = 45)		
Outcome Measures						
	Baseline	6 months	12 months	Baseline	6 months	12 months
MANSA – mean (SD)						
Overall summary (mean) score *(from 1 to 7*)	4.4 (0.3)	4.5 (0.4)	4.7 (0.2)	4.2 (0.2)	4.3 (0.1)	4.3 (0.3)
HAS – mean (SD)						
Overall summary mean score (*from 1 to 10*)	6.2 (0.6)	6.6 (0.6)	7.0 (0.8)	6.2 (0.2)	6.3 (0.5)	6.7 (0.2)
EssenCES – mean (SD)						
Patient cohesion sum score (*from 0 to 20*)	10.1 (0.7)	8.8 (1.0)	9.3 (0.7)	10.0 (0.5)	10.6 (0.2)	9.3 (0.7)
Experienced safety sum score (*from 0 to 20*)	12.7 (1.4)	15.4 (1.2)	16.3 (2.4)	10.1 (1.5)	16.3 (2.3)	15.4 (2.7)
Therapeutic hold sum score (*from 0 to 20*)	12.4 (1.0)	10.7 (1.5)	11.6 (1.2)	12.1 (0.1)	11.7 (1.0)	12.2 (0.5)
FSS – mean (SD)						
Overall summary mean score (*from 1 to 5*)	3.2 (0.2)	3.3 (0.2)	3.3 (0.3)	3.2 (0.0)	3.3 (0.1)	3.3 (0.1)
QPR – mean (SD)						
Intrapersonal sum score (*from 17 to 85*)	65.4 (1.7)	66.4 (2.0)	65.6 (1.0)	62.6 (4.1)	64.1 (2.0)	63.9 (1.1)
Interpersonal sum score (*from 5 to 25*)	19.1 (0.3)	18.9 (0.4)	18.9 (0.7)	18.9 (0.8)	19.0 (0.7)	19.7 (0.9)
MBI – mean (SD)						
Professional Efficacy mean score (*from 0 to 6*)	5.2 (0.3)	5.3 (0.2)	5.0 (0.1)	5.2 (0.0)	5.3 (0.0)	5.1 (0.0)
Exhaustion mean score (*from 0 to 6*)	2.3 (0.6)	2.4 (1.1)	2.2 (0.8)	2.2 (0.6)	2.8 (1.1)	2.4 (0.3)
Cynicism mean score (*from 0 to 6*)	1.4 (0.3)	1.2 (0.2)	1.5 (0.9)	1.5 (0.2)	1.7 (0.7)	1.8 (0.2)

### Withdrawal rates, completion rates and monitored 1:1 sessions

5 patients in the intervention group (9 %) and 11 (19 %) in the control group were lost to follow up at 6 or 12 months. The majority (11) of these patients reported they were no longer interest in being involved. However, one participant in the intervention group had been deported and another had been sent back to prison and unable to be located in the prison system, while three participants in the control group had been sent back to prison. The probability of being lost to follow up at 6 months/12 months appears to be potentially related to some of the baseline characteristics and in particular gender; with the odds of being lost to follow up for women estimated to be 2.2 times the odds of men (OR: 2.2; 95 % CI: 0.5 to 9.2). Therefore, for gender, the between intervention and control group differences in those lost to follow up at 6 months/12 months tend to be larger than the chance differences observed at baseline. However, the differential attrition did not appear to lead to imbalance in gender amongst those patients remaining in the trial (i.e. not lost to follow up) compared with all patients at baseline. Both age and mental health act status seem more or less balanced both at baseline and amongst those lost to follow up at 6 months/12 months. For length of stay and clinical diagnosis, the between intervention and control group differences in those lost to follow up at 6 months/12 months tend to be larger than the chance differences observed at baseline. However, the differential attrition does not appear to lead to imbalance amongst those patients not lost to follow up compared with all patients at baseline.

The number of patients from those randomised completing the primary outcome (MANSA) scale at 6 months was 48 (87 %) of the intervention group and 42 (74 %) of the control group. At 12 months, the numbers were 47 (85 %) of the intervention group and 42 (74 %) control group. The rate for the other patient outcomes ranged from 46 (84 %) to 48 (87 %) for the intervention group at 6 months and between 41 (72 %) and 42 (74 %) for the control group. At 12 months, the numbers completing the outcomes in the intervention group were 47 (85 %) for all of the assessments and between 39 (68 %) and 42 (72 %) in the control group. It appears the majority of patients completed the primary and secondary outcomes at 6 and 12 months. The number of nurses included in the analysis was much lower with 60 % of both the intervention group and control group completing the assessment at 6 months and 24 (51 %) of the intervention group and only 18 (40 %) of the control group completing the assessment at 12 months.

The number of patients giving any data at 6 months, and the number of patients giving any data at 12 months, is similar for each of the outcomes/subscales. Similarly, the number of nurses giving any data at 6 and 12 months is similar for each of the outcomes/subscales. This suggests that there is not an outcome/subscale that participants did not want to complete at all more than others. Amongst those who completed an outcome measure at all, the mean proportion of the measure completed was high for all outcomes, mostly being between 90 % and 100 %. This suggests that completeness of outcomes would not be a significant issue for the main trial.

The mean number of 1:1 monitored sessions between nurses and patients per month was 2 for each month for those on the intervention, but was 3 each month for those on the control. Considering the DIALOG sessions are included in the count towards the 1:1 sessions for those on the intervention as well, this suggests that patients on the intervention received on average fewer 1:1 sessions with the nurse than the control patients and that the DIALOG intervention does not create an increased workload for the service. The duration of the sessions were not recorded.

### Participant experiences

Both patient and nurses involved in the intervention expressed broadly positive views about the approach.

A numbers of patient comments centred on the fact that the approach gave a structure to their meetings with nurses, was focussed on goals, and was an opportunity to reflect on issues in their lives and to think about making changes such as in the quote underneath:

*“You got used to it after one or two sessions you know. It was easier to communicate and talk about things and that”.*

Some patients were also able to identify specific changes which were beneficial for them from the intervention (for example, getting a job volunteering). They also liked using iPads and how the process eased communication with the person doing the intervention. The financial reward for undertaking outcome assessments was also a strong incentive.

In terms limitations of the intervention, some felt that concerns raised during the sessions were not taken seriously by staff. This linked to the theme most often brought up by participants – the importance of the relationship to the staff member doing the study.

Nurses identified some challenges relating to the initial difficulties encountered in arranging some sessions with their patients due to difficulties such as unsettled ward environments or low staffing levels. Having protected time for the delivery of the session was seen as beneficial to the smooth running of the sessions as was arranging meetings to fit into daily running of the ward. For some nurses, working with new technology and software was difficult and there was some uncertainty in the initial sessions as to whether they had used the iPads correctly. However, the majority of nurses were knowledgeable about using iPads, and found using these tablets and software was straight forward and easy to use while those who were initially anxious about using the software became more confident in its use as more sessions took place.

The use of a structured approach and SFBT was viewed positively as noted below and using the structured communication approach was viewed as beneficial from both a practical and professional perspective.

*“Solution Focused Approach isn’t a hat I put on when I do my sessions. The process is happening naturally and in my every day interactions with patients”.*

## Variability of the outcomes of interest

### Outcomes

Data was collected from July 2012 to January 2015. The outcomes at baseline, 6 months and 12 months are shown in Table 2 while the estimated treatment effects and corresponding confidence intervals calculated for all outcomes measured at 6 and 12 months are shown in Table 3. Table 4 records the number of participants completing outcome assessments at 6 months and 12 months. Since this is a pilot study where no formal sample size calculation was done, and a cluster randomised trial with only 6 clusters, the study is underpowered to be able to detect statistically significant differences. Eldridge and Kerry [26] suggest that it is important to use the limits of confidence intervals to judge any likely effect, not the effect estimate itself or a *p*-value.

**Table 3:** The estimated treatment effects and confidence interval for all outcomes

	Treatment effect (intervention – control) and confidence interval	
Outcome Assessment		
	6 months	12 months
MANSA	0.2 (−0.4 to 0.8)	0.4 (−0.3 to 1.1)
HAS	0.3 (−0.9 to 1.6)	0.3 (−1.0 to 1.7)
EssenCES		
Patient Cohesion	−1.7 (−3.3 to −0.2)	0.0 (−1.6 to 1.5)
Experienced Safety	−0.9 (−5.1 to 3.2)	0.9 (−4.9 to 6.6)
Therapeutic Hold	−1.1 (−3.9 to 1.8)	−0.6 (−2.8 to 1.6)
FSS	0.0 (−0.3 to 0.3)	0.0 (−0.5 to 0.5)
QPR		
Intrapersonal	2.2 (−2.3 to 6.7)	1.7 (−0.7 to 4.1)
Interpersonal	−0.1 (−1.3 to 1.2)	−0.9 (−2.7 to 1.0)
MBI		
Professional Efficacy	0.1 (−0.3 to 0.4)	−0.1 (−0.3 to 0.1)
Exhaustion	−0.4 (−2.8 to 2.0)	−0.2 (−2.0 to 1.7)
Cynicism	−0.5 (−1.6 to 0.5)	−0.4 (−2.4 to 1.7)

**Table 4:** No. (% of those randomised) of participants included in each analysis

	6 months		12 months	
	Intervention (*n* = 55, k = 47)	Control (*n* = 57, k = 45)	Intervention (*n* = 55, k = 47)	Control (*n* = 57, k = 45)
MANSA				
Overall mean score	48 (87)	42 (74)	47 (85)	42 (74)
HAS				
Overall mean score	45 (82)	41 (72)	47 (85)	41 (72)
EssenCES				
Patient cohesion	47 (85)	41 (72)	47 (85)	41 (72)
Experienced safety	47 (85)	41 (72)	47 (85)	40 (70)
Therapeutic hold	47 (85)	41 (72)	47 (85)	41 (72)
FSS				
Overall mean score	47 (85)	42 (74)	47 (85)	41 (72)
QPR				
Intrapersonal	48 (87)	42 (74)	47 (85)	41 (72)
Interpersonal	48 (87)	41 (72)	47 (85)	39 (68)
MBI				
Professional Efficacy	28 (60)	27 (60)	24 (51)	18 (40)
Exhaustion	28 (60)	27 (60)	24 (51)	18 (40)
Cynicism	28 (60)	27 (60)	24 (51)	18 (40)

For the primary outcome at 6 months, the difference in the overall summary mean MANSA score is estimated to be as much as 0.8 higher in the intervention group to as much as 0.4 lower, and at 12 months is estimated to be as much as 1.1 higher in the intervention group to as much as 0.3 lower. This indicates the likely extreme boundaries of effect in the main trial, and that a positive effect of the intervention is not ruled out. A full trial would be justified to estimate the effect with greater certainty.

Similar conclusions can be drawn for each of the secondary outcomes with only one outcome excluding a null effect (the EssenCES subscale - Patient cohesion sum score at 6 months). However, it should be noted that it would expected to see the null effect ruled out in 5 % of cases just by chance due to the multiple outcomes that are being examined.

For the primary outcome, the standardised effect size (treatment effect divided by standard deviation) and confidence interval at 6 and 12 months are 0.7 (−0.9 to 2.4) and 1.4 (−0.4 to 3.1), respectively. The confidence intervals do contain 0.3, where 0.3 is the sort of standardised effect size that might be expected for an effective complex intervention and the sort of standardised effect size that investigators often power their trials on. The estimate of the ICC for the primary outcome at 6 and 12 months is 0.04 (0.00 to 0.17) and 0.05 (0.00 to 0.18). The standard deviation is 0.3 at both time points.

### Costs

The results of the cost-consequences analysis for the intervention and control groups are presented in Table 5. The average cost of stay in the facility was calculated by multiplying the number of days by the mean cost of bed-day for the units (£486.90). The average cost of treatment over the 12 month period (including the cost of stay in the facility, cost of incidents and cost of intervention) was in the range £167,049–£167,378 for the intervention group and £165,491–£166,282 for the control group. The total cost of the intervention was £30,413 including additional time to deliver the intervention and £29,100 excluding additional time (£545 and £529 per patient).

**Table 5:** Cost-consequence analysis

Resource Use and Costs	Intervention group (*n* = 55)	Control group (*n* = 57)
Total cost of intervention	£30,413	£0
Cost of intervention per patient including nurse training	£529–576	£0
Average number of days in the facility over 12 months, mean (SD)	341 (56)	338 (37)
Average cost of stay in the facility (bed-day cost) over 12 months	£166,064	£164,506
Number of incidents over 12 months		
suicide attempt	2	17
self-harm	47	93
violence against others	50	96
violence against inanimate objects	81	76
absconding/escape	8	11
Cost of incidents over 12 months^a^		
suicide attempt	£1,688–£3,622	£14,350–£30,788
self-harm	£7,949–£12,961	£15,729–£25,647
violence against others	£3,323–£10,954	£6,380–£21,033
violence against inanimate objects	not available	not available
absconding/escape^b^	£10,737–£10,816	£14,763–£14,872
Total cost of incidents	£23,697–£38,354	£51,222–£92,340
Cost of incidents per patient	£456–£738	£985–£1,776
Average treatment cost (intervention + stay + incidents)	£167,049–£167,378	£165,491–£166,282

^a^estimated using micro-costing approach

^b^assumes 50 % absconding 50 % escape, since these incidents were recorded together

The levels of disturbance are shown in Table 6. During the three month period before the structured communication approach was initiated there were a similar number of episodes of disturbed behaviour recorded in the majority of categories in both groups. The exceptions were higher numbers of hours in seclusion and suicide attempts in the control group and a higher number of violent attacks on inanimate objects and incidents of abusive/racial language in the intervention group. During the following 12 months, following the commencement of the structured communication approach, the intervention group recorded less overall disturbed behaviour compared to the control group. This included a lesser number of seclusions (9 vs.37), hours of seclusion (328 vs. 758), physical restraints (22 vs. 35), attempts to self-harm (47 vs. 93) and violent acts against others (50 vs. 96). The numbers of incidents were higher in the control group compared to the intervention group, although these were not compared statistically. The total cost of incidents was £23,697–£38,354 for the intervention group and £51,222–£92,340 for the control group. The most costly incidents were escapes (£2,240–£2,250) since they involved police investigation, followed by suicide attempts (£844–£1,811) which incurred inpatient costs.

**Table 6:** Level of disturbance

	Intervention clusters (*n* = 55)		Control clusters(*n* = 57)	
Disturbance (Number of)				
	Pre-ComQuol	Post-ComQuol	Pre-ComQuol	Post- ComQuol
Seclusions	11	9	9	37
Hours of seclusion	231	328	150	758
Physical restraint	8	22	8	35
Suicide attempts	1	2	7	17
Self-harm attempts	19	47	10	93
Violent acts on others	21	50	23	96
Violent attacks on inanimate objects	48	81	15	76
Attempted absconding/escapes	3	2	1	7
Actual absconding/escapes	1	8	4	11
Abusive/racial language	201	362	94	313

## Discussion

### Feasibility of recruitment

Recruitment to studies involving forensic mental health care is extremely difficult [27] resulting in limited large scale studies in this area. Although there were challenges in gaining access to participants in this study due to the procedures required by individual units prior to access to the clinical areas being granted, the study recruited sufficient numbers of staff and patients to be included in the study. The process of liaising with individual units to agree on the best strategy seemed to be the best way of recruiting participants. The wide inclusion criteria helped to secure a wide pool of participants In addition, there did not seem to be any identified population where there were difficulties in recruiting to the study. The three intervention sites adopted different approaches for identifying nurses to be recruited to the study. In a recent study using a similar method, Priebe and colleagues [28] suggest this pragmatic approach is a key strength of the study as the intervention is rolled out as it would be in practice therefore the results are likely to be generalisable to other forensic medium secure services.

### Feasibility, quality and acceptability of interventions

It is important that the intervention is reproducible in a large scale trial. The training programme, SFBT manual and monthly meetings between research team and nurses provided a strong base for ensuring a consistent approach was adopted by all the nurses involved in the intervention. The training programme was designed with the perception that the nurses would not have prior experience of the DIALOG approach or Solution Focused Brief Therapy. The evidence from the taped sessions and session records suggests the nurses were able to deliver the intervention as instructed allaying concerns about the quality of the intervention provided by clinicians untrained in SFBT.

The majority of nurses randomised to the intervention group attended the training session (35 out of 47 (74 %). These were well received by the nurses as noted in the qualitative interviews. Few patients (*n* = 7, 13 %) and nurses (*n* = 6, 13 %) withdrew from the intervention. The qualitative evidence suggests both the nurses and patients viewed the intervention as valuable and able to be understood and followed by nurses and patients. The structure of the approach also allowed discussions to be structured and centred on areas considered important by patients. Approximately a quarter of nurses recruited to the study did not attend the training sessions. As noted earlier, part of the reason for the non-attendance was due to delays in the period between recruitment and undertaking the training, particularly for the first cohort. The subsequent cohort of nurses had a much shorter time span between recruitment and training with the result that the proportion of recruited nurses attending the training was much higher. Those nurses in the 2nd and 3rd intervention were also given a clearer explanation as to what involvement in the study would entail which may have also helped nurses be more confident about their role and commitment. This policy will be maintained in the proposal for a full scale trial. However, some delay between recruitment and the delivery of the training is inevitable as the service can only organise their work roster for staff to attend the training sessions once randomisation has taken place. It was also noted that nurses became more proficient in their use of DIALOG and SFBT as they undertook more sessions. It may be preferable to allow nurses some space to practice their skills before commencing the interventions.

The frequency and duration of the sessions was guided by the previous work of Priebe and colleagues (Priebe et al., 2007, Priebe et al, 2013) as well as a number of practical considerations underpinning the scheduling of sessions. There were a number of competing pressures from nursing related issues (shift patterns, staffing numbers), patient related issues (therapeutic programme, mental state), as well as the availability of rooms at a convenient time to undertake the session. These pressures were acknowledged in discussions between some nurses and the Research Assistant. If more frequent/intensive interventions were to be considered, it is likely that other practitioners such as occupational therapists, social workers, psychologists and doctors as well as health care assistants should be considered as facilitators of the sessions as long as they were able to attend the training sessions and to commit to undertake the sessions for the agreed frequency and duration of the intervention

In terms of the acceptability of the intervention to the patients, although there were many positive comments about the strengths of the intervention, some limitations were also noted. Some patients complained that nurses did not do anything about the patients' concerns. Two main reasons seemed to be underpinning this view. Firstly, some of the problems were unable to be changed whilst being in a forensic unit. The two most common areas where help was requested were in relation to medication and their job situation. There are limitations to the amount of change that could take place in these areas in the inpatient environment. However, the most frequent view from the patients was that they felt that it was the attitude of the nurse doing the intervention which determined whether they found it helpful or unhelpful. The centrality of staff-patient relationships to quality of life on the forensic units and a sense of fairness, safety, humanity and trust was also reported as important. In a definitive trial, it may be helpful to formally present this feature of the therapeutic relationship as part of the training programme before commencing the intervention.

The study demonstrated that a full scale cluster randomised trial to examine the effectiveness of a structured communication approach using DIALOG supported by Solution Focused Brief Therapy was feasible. For the primary outcome at 6 months, the difference in the overall summary mean MANSA score is estimated to be up to 0.8 higher in the intervention group or 0.4 lower and as much as 1.1 higher in the intervention group or 0.3 lower at 12 months. The estimated treatment effect of the primary outcome is clinically important and the estimated overall summary mean MANSA scores indicate a positive effect of the intervention is not ruled out. Similar conclusions can be drawn for each of the secondary outcomes. However, as expected given the small sample size, particularly the number of clusters, there are no firm conclusions to be drawn from these estimates about the effectiveness of the intervention. The intervention is promising but there is a large degree of uncertainty which makes a larger trial essential.

The estimate of the ICC for the primary outcome at 6 and 12 months is 0.04 (0.00 to 0.17) and 0.05 (0.00 to 0.18). The standard deviation is 0.3 at both time points. It is difficult to triangulate these with similar studies to form an estimate of the ICC that could be used in the main trial sample size calculation due to a lack of studies examining quality of life in medium secure units. A more conservative estimate of 0.07 or 0.08 would be employed for a main trial.

The pilot study also provided estimates of intervention costs, the cost of incidents and the cost of stays in a medium secure mental healthcare facility to inform the future study. The study identified resources which are likely to be affected by the intervention, and the major sources of uncertainty associated with use of these resources. Health economics analysis showed that incidents are costly, since they are associated with significant use of NHS resources and police. Due to resource constraints, a micro-costing of incidents based on informed clinical opinion was collected and analysed rather than collecting patient-level data on resource use by patients. A number of incidents in the same category (i.e. violent acts against others) are likely to result in markedly different resource use therefore it would be helpful for the resources required for each individual incident to be recorded separately in a definitive trial.

### Strengths and weaknesses

This pilot cluster randomised trial was designed to test the feasibility of a full trial and to optimise the intervention and trial approach. It has provided an initial indication that the intervention has the potential to be effective but the small sample size means it did not have the power to detect significant clinical differences. The health economic data gave a clear overview of the costs involved in the study. However, it was acknowledged that collecting individual patient data may be beneficial in a full trial particularly in relation to the costs associated with disturbed behaviour. Looking at what individual costs are associated with each episode of disturbance would give a clearer indication of the costs accrued following a disturbed event. Examining the incident costs for longer than 12 months following the start of the intervention may also give a better indication of on-going costs.

There were proportionally more women patients who withdrew from the study than men. This may be associated with the specific sample of patients recruited for the pilot study. However, it would be beneficial to look more closely at any reasons for higher dropout rates for women and also whether it may be helpful to offer some ongoing support during the intervention. The number of nurses lost to follow up indicates some uncertainty of the value of the findings of the Maslach Burnout Inventory. It is worth noting that only 6 (13 %) nurses actively withdrew from providing the intervention. However, the number who did not complete the outcome assessments at 6 and 12 months was much higher. This questions the value of including nursing outcomes in a full trial. Alternatively, procedures may need to be put in place during the trial to try to reduce missing data.

No information was collected on the ratings of need for therapeutic security for the patients. As this was a pragmatic trial, the team determined that clinicians would be making judgements on the relevance of the need for medium secure mental health care based on the admission criteria for these units enshrined in the standardised specifications which accompany contracts. All patients recruited to the study would have had an HoNOS-Secure [29] and using HoNOS-Secure or the DUNDRUM-triage security scale [30] may be a useful additional measure in any full trial.

The collection of qualitative data was viewed as a positive addition to the quantitative data. It helped gain a greater understanding of the intervention’s feasibility and acceptability and also provided an opportunity for improvements in the intervention and conduct of the trial. One recommendation would be that any qualitative/focus group data be collected within 2 months of the trial finishing in a full trial. In addition, the centrality of staff-patient relationships to quality of life on the forensic units and the importance of a sense of fairness, safety, humanity and trust as important factors in this relationship could be made more central to the intervention and factored in to any measurement of quality of life on forensic wards similarly to how prison research has focused on these factors with regards to staff-prisoner relations, the quality of prison life and levels of distress [31].

## Conclusion

The trial design appears viable as the basis for a large full-scale trial. The procedures seem to function well; randomisation procedures, intervention approach (training and application) while the measures used were understood by the participants and gave relevant outcome information. The response rates were good with low patient withdrawal rates. The qualitative responses also suggest general satisfaction with the approach.

The variability of the outcomes from this pilot study provides a starting point for considering the inputs for a sample size calculation for a main trial. Further data are needed to ensure the robustness of the estimate of the variation. The estimated treatment effect of the primary outcome is clinically important, and a positive effect of the intervention is not ruled out so a full trial is justified to estimate the effect with greater certainty.

Health economics analysis showed that incidents are costly, since they are associated with significant use of NHS and police resources. The real cost of incidents may be even higher when analysed using patient-level data. Examining the incident costs for longer may give a better indication of on-going costs. There is also a need to look at the reasons for higher dropout rates for women and whether to offer ongoing support. An examination of ratings of need for therapeutic security may be useful in any future study as well the possibility of other practitioners being involved in facilitating the intervention. Finally, the number of nurses withdrawing from the study and not undertaking the outcome assessments indicates some uncertainty of the value of including nursing outcomes in a full trial.

## Abbreviations

ComQuol: Structured communication approach on quality of life
EssenCES: Essen climate evaluation schema
FSS: Forensic satisfaction scale
HAS: Helping alliance scale
HoNOS-Secure: Health of the nation outcome scales for users of secure and forensic services
ICC: Intra-cluster correlation coefficient
MANSA: Manchester short assessment of quality of life
MBI: Maslach Burnout inventory
PCTU: Pragmatic clinical trials unit
QPR: Process of recovery questionnaire
RfPB: Research for patient benefit
SFBT: Solution focused brief therapy
SF-SUS: Secure facilities service use schedule
